# High Dose Thiotepa in Patients with Relapsed or Refractory Osteosarcomas: Experience of the SFCE Group

**DOI:** 10.1155/2014/475067

**Published:** 2014-02-04

**Authors:** Perrine Marec-Berard, Céline Segura-Ferlay, Marie-Dominique Tabone, Helene Pacquement, Cyril Lervat, Jean-Claude Gentet, Claudine Schmitt, Nathalie Gaspar, Laurence Brugières

**Affiliations:** ^1^Institute for Paediatric Haematology and Oncology, Leon Bérard Cancer Centre, University of Lyon, 1 Place Joseph Renaut, 69008 Lyon, France; ^2^Unit of Biostatistics, Leon Bérard Cancer Centre, University of Lyon, 69008 Lyon, France; ^3^Department of Paediatric Haematology and Oncology, Hôpital Armand Trousseau, 75012 Paris, France; ^4^Department of Paediatrics, Curie Institute, 75248 Paris, France; ^5^Department of Paediatrics, Children's Hospital, 59037 Lilles, France; ^6^Department of Paediatrics, La Timone Hospital, 13385 Marseille, France; ^7^Department of Paediatrics, Children's Hospital, 54000 Nancy, France; ^8^Department of Paediatrics, Gustave Roussy Institute, 94805 Villejuif, France

## Abstract

*Introduction*. Osteosarcoma relapse has a poor prognosis, with less than 25% survival at 5 years. We describe the experience of the French Society of Paediatric Oncology (SFCE) with high dose (HD) thiotepa and autologous stem cell transplantation (ASCT) in 45 children with relapsed osteosarcoma. *Patients and Methods*. Between 1992 and 2004, 53 patients received HD thiotepa (900 mg/m^2^) followed by ASCT in 6 centres. Eight patients were excluded from analysis, and we retrospectively reviewed the clinical radiological and anatomopathological patterns of the 45 remaining patients. *Results*. Sixteen girls and 29 boys (median age, 15.9 years) received HD thiotepa after initial progression of metastatic disease (2), first relapse (26), and second or third relapse (17). We report 12 radiological partial responses and 9 of 31 histological complete responses. Thirty-two patients experienced further relapses, and 13 continued in complete remission after surgical resection of the residual disease. Three-year overall survival was 40%, and 3-year progression-free survival was 24%. Delay of relapse (+/− 2 years from diagnosis) was a prognostic factor (*P* = 0.011). No acute toxic serious adverse event occurred. *Conclusion*. The use of HD thiotepa and ASCT is feasible in patients with relapsed osteosarcoma. A randomized study for recurrent osteosarcoma between standard salvage chemotherapy and high dose thiotepa with stem cell rescue is ongoing.

## 1. Introduction

Over the last 30 years, the prognosis of children and adolescents with localised osteosarcomas has improved to reach current overall survival (OS) of around 60% following an intensive chemotherapy regimen that includes high dose methotrexate, ifosfamide, etoposide, adriamycin, and cisplatin [1]. However, the prognosis of patients relapsing or presenting with metastatic disease at diagnosis is very poor, with 3-year OS of 10 to 50% and relapse-free survival (RFS) of 10 to 20% [2–7]. Therefore, the development of new therapeutic strategies for these patients is warranted.

High dose chemotherapy (HDCT) with peripheral stem cell support has been evaluated for the treatment of many high risk paediatric tumours [8–11]. However, few publications report the results of this approach in the treatment of relapsed osteosarcomas. Moreover, most of these reports are based on small numbers of patients and heterogeneous therapeutic modalities [7, 12–14].

Thiotepa (N N′ N′′ triethylenethiophosphoramide; TTP) is an alkylating agent of the ethyleneimine family that is known for its antitumour effect on many types of malignancies [15]. Its efficacy against osteosarcomas has been suggested in a previous study of the French Society of Paediatric Oncology (Société Française du Cancer de l'Enfant; SFCE) [16] but remains to be demonstrated in a larger number of patients.

We report the experience of the SFCE group with relapsed or refractory osteosarcomas treated with high dose TTP with stem cell support.

## 2. Patients and Methods

### 2.1. Patients

Between 1992 and 2004, 53 patients with relapsed or refractory osteosarcomas were treated with high dose TTP (900 mg/m^2^) followed by reinjection of autologous peripheral blood stem cells (PBSC). Patients were identified from either pharmacy records or institutional databases in 6 different institutions of the SFCE Bone Tumour Group. We excluded from analysis 8 patients who received high dose thiotepa as a first-line consolidation treatment for metastatic disease and retrospectively reviewed the medical charts of the 45 remaining patients. The French Osteosarcoma Committee approved this project.

### 2.2. Treatment

**Figure 1:**
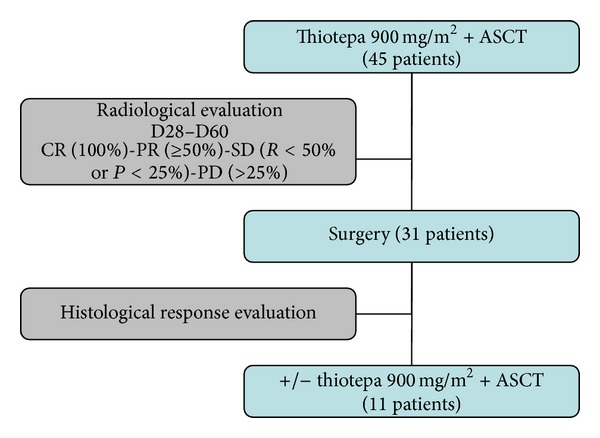
Treatment schedule.

Figure 1 summarizes the treatment schedule. Patients diagnosed with relapse or progression of osteosarcoma underwent treatment with or without conventional chemotherapy according to the practices of each centre and in accordance with the French recommendation for the treatment of recurrent osteosarcomas published in 1994 [7]. The French strategy for managing these patients is influenced by the response of the primary tumour to chemotherapy. Good responders usually receive a second-line chemotherapy indicated for poor responders, and initial poor responders with multiple or unresectable metastases are treated with various second-line chemotherapy regimens in an attempt to allow complete surgery. Those with a solitary nodule or an easily resectable lesion usually do not receive adjuvant treatment. Some undergo intensification with TTP, often because of an apparent chemosensitivity.

PBSC harvesting by leukapheresis was performed according to each centre's standard procedure. Granulocyte colony-stimulating factor (G-CSF) was used for PBSC mobilization either in steady state (27 patients) or following myelosuppressive chemotherapy (18 patients).

Thiotepa was administered as a 2-hour intravenous infusion, at a dose of 300 mg/m^2^/d for 3 consecutive days. PBSC were reinjected 48 hours after the last dose of TTP (D0). G-CSF was recommended from day 5 after PBSC reinjection until 2 consecutive absolute neutrophil counts at 48 h intervals exceeded 0.5 × 10^9^/L. Supportive care measures were taken according to each centre's local procedure. Hospitalisation was recommended from the day of TTP administration to the end of neutropenia.

Surgery was recommended whenever possible, 30 days after the course of chemotherapy.

### 2.3. Response Evaluation

A computed tomographic (CT) scan was performed between 28 and 60 days following the first intensive thiotepa administration. All images were centrally reviewed to assess the radiological tumour response. Only patients with measurable disease were considered evaluable for response. Measurable disease was defined as the presence of at least one lesion that could be accurately measured in at least one dimension, which corresponded to the longest dimension greater than 20 mm with conventional techniques or greater than 10 mm using spiral CT. The sum of the products of the 2 greatest perpendicular diameters of each measurable lesion was used to estimate the radiological response. Complete response (CR) was defined as the disappearance of all measurable tumours for more than one month; partial response (PR) corresponded to a reduction greater than 50%. Stable disease was defined as an increase of less than 25% or a reduction of less than 50%, and progression of disease was defined as an increase of greater than 25% or the appearance of new lesions.

A pathologist centrally reviewed histological reports of all patients who underwent surgery and assessed and classified histological responses into 2 groups. Good histological response was defined as no residual tumour, predominant fibrosis, and osteoid matrix with no or exceptional visible viable cells. All other cases were classified as having a poor histological response.

### 2.4. Statistical Analysis

Overall survival was calculated from the first day of treatment with TTP to the date of death from any cause or to last follow-up for living patients (censored observation). Survival estimates were calculated using the Kaplan-Meier method [17]. Differences in survival estimates between groups were assessed by Log-Rank test [18]. Median follow-up was calculated using reverse Kaplan-Meier estimation. Progression-free survival (PFS) was measured from the date of beginning of treatment to the date of disease progression or death or censored at last follow-up.

## 3. Results

### 3.1. Patient Characteristics

We evaluated 45 patients (16 female, 29 male; median age 15.9 years, range from 4 to 31 years).  Table 1 shows patient characteristics at the time of initial diagnosis. Initial disease was in the lower limb in 36 patients, in the upper limb in seven, and axial in two. Disease was localised in 33 patients. All patients received chemotherapy as part of their initial treatment, which consisted of either paediatric schedules based on high dose methotrexate and adriamycin or etoposide-ifosfamide combinations [19] or adult-type schedules without high dose methotrexate. All patients underwent surgery of the primary tumour. Based on Huvos grading [20], histological response to initial chemotherapy was good in 17 patients and poor in 28.  Table 2 shows patient characteristics and disease status when they received high dose TTP. Disease progressed in 2 patients after initial treatment; 26 patients were in first relapse with a median time from diagnosis to first disease recurrence/progression of 18.2 months, and 17 patients were in further relapse.

Relapse localisations were isolated lung metastasis in 23 patients, isolated bone site in one, combined lung and another localisation in 15, and other sites in four. At the time of relapse diagnosis, all sites were considered resectable in 20 patients and inaccessible to surgical resection in 23 patients. Data are missing for 2 patients.

Thirty-one patients underwent surgery, 23 had complete resection and eight had partial resection. Eleven patients had a second course of high dose TTP after surgery. A total of 66 courses of 900 mg/m^2^ TTP followed by PBSC rescue were administered, and no major or unexpected toxicity is reported. No death related to high dose TTP was documented and no patient experienced life-threatening extrahaematologic severe toxicity.

### 3.2. Responses and Survival

Six patients were not evaluable for radiological response because high dose TTP was administered after surgery.

Among the 39 remaining patients, we observed PR in 12, stable disease (SD) in 18, and progressive disease (PD) in nine. Thirty-one patients had subsequent surgery. Among them, 24 underwent wide resection of all localisations, and histological response was good in nine and poor in 22. Thirty-two patients experienced further relapse, and a second CR was obtained in 13 of them. The median time to PFS was 8.8 months (95% confidence interval (CI 95%), 2.3 to 15.3).

At the time of analysis, the median follow-up was 133.7 months (range from 66.3 to 205.7). Twelve of the 45 patients (27%) were still alive, and the median OS was 21.2 months (CI 95%, 13.4 to 8.9). All survivors could have surgical resection of all tumour sites.

**Figure 2:**
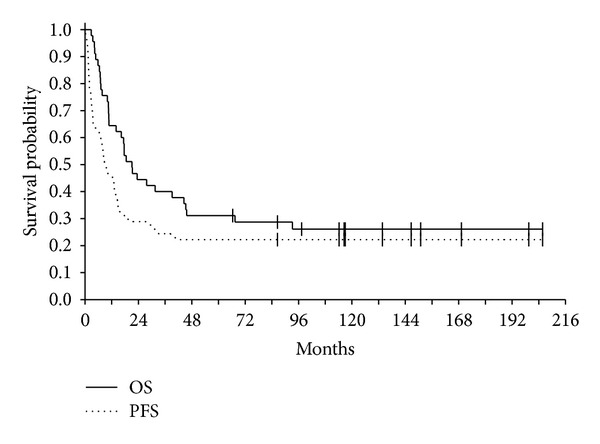
Progression-free (PFS) and overall (OS) survival of 45 patients.

Three-year OS was 40% (CI 95%, 27 to 55), and 5-year OS was 31% (CI 95%, 20 to 46). Three-year PFS was 24% (CI 95%, 14 to 39), and 5-year PFS was 22% (CI 95%, 13 to 36) (Figure 2).

In univariate analysis, delay of relapse (+/− 2 years from diagnosis) was the only prognostic factor (*P* = 0.011). No significant impact on survival was observed for initial histological response, radiological response to TTP, surgical resection, histological response to TTP, and the number of previous relapses (Table 3).

## 4. Discussion

Over the past 30 years, the prognosis of localised osteosarcoma has significantly improved. At initial diagnosis, age, axial localisation, metastatic status, and tumour size have been identified as prognostic factors in addition to histological response and surgical margins [21, 22]. For metastatic diseases, the quality of surgery and number of metastases have prognostic value [6, 21].

However, approximately 30 to 40% of patients will relapse, mostly within the first 3 years from diagnosis, and their prognosis is poor, with 5-year OS between 20 and 28% [3].

Attempts have been made to improve survival in this population. Increasing chemotherapy dose induced short-lasting remission but did not increase survival. High dose chemotherapy with carboplatin, etoposide, ifosfamide, and high dose cyclophosphamide yielded only 28% response [23]. Results of further investigations with HCT in relapsed osteosarcomas with short remissions and early relapses have been disappointing [7, 24]. The Italian experience in metastatic relapses of osteosarcomas with an association of high dose carboplatin and etoposide showed a progression rate of 24.4%, with 20% 3-year OS and 12% disease-free survival [12]. Reporting on their experience with high dose carboplatin, etoposide, thiotepa, or cyclophosphamide, Sauerbrey and colleagues found no benefit of high dose strategy compared to conventional treatments in this indication [14].

Miniero and colleagues reported the feasibility of combining high dose carboplatin and etoposide in a series of 6 patients with relapsed osteosarcomas, but the study size was too small to demonstrate a benefit in terms of survival [25].

In the experience of the European Group for Blood and Marrow Transplantation (EBMT) with 149 patients with osteosarcomas, 5-year survival was 33% in those patients who received high dose chemotherapy in first-line treatment (55 patients) and 14% in those treated with high dose after relapse (77 patients) [26]. Based on these results, the EBMT concluded that there was not enough evidence for the use of high dose chemotherapy in osteosarcoma treatment.

Thiotepa is an alkylating agent with antitumour activity that has been used for 30 years to treat adult ovarian [27] and breast [28, 29] cancers. The dose-limiting toxicity of this drug is notably on the gastrointestinal tract (mucositis and diarrhoea) and the central nervous system (drowsiness and seizure).

The first Phase I study in children has established a maximum tolerated dose (MTD) of 65 mg/m^2^ every 21 days. However, Phase II studies conducted at these doses in children showed no significant efficacy [30].

Thiotepa has pharmacokinetic properties that favour the use of dose intensification. With reinjection of stem cell rescue to overcome its myelotoxicity, the MTD of TTP has been multiplied 15 times to reach 900 mg/m^2^ in adults and children [31–33]. In children aged 2 to 12 years, similar pharmacokinetic characteristics were observed with thiotepa at 900 mg/m^2^ and 75 mg/m^2^ [31]. Rodenhuis and colleagues have also shown acceptable toxicity giving 2 or 3 courses of high dose TTP at 5-week intervals [34].

Thiotepa has been the subject of several investigations in solid tumours in children [16, 35]. In the Phase II trial conducted by the SFCE group in 1987, children with refractory or relapsed solid tumours received a single course of TTP 900 mg/m^2^ followed by stem cell rescue. Among the 7 osteosarcomas treated, 4 radiological responses were reported. Assessment of histological response in 5 cases yielded percentages of viable cells of 0, 5, 20, 20, and 90% [16]. However, the median duration of these responses was only 7 months, which was quite similar to previous published data.

The radiological response rate reported here (31% objective response, 46% stable disease) allows us to conclude that high dose TTP is effective in the treatment of osteosarcoma. Because most relapses involved the lungs, the histological response to chemotherapy is difficult to assess and classify. Huvos grading [20] is not applicable, and we decided to classify patients as good responders only if they had no residual tumour, predominant fibrosis, and osteoid matrix with no or exceptional visible viable cells. Using this standardized grading in the central review process, we found 29% good histological responses, which is probably underestimated. From both the radiographic and histological responses, these results provide evidence that TTP could be considered an active drug for the treatment of osteosarcomas.

One limitation of this study is that patient selection probably impacted the outcomes reported. Indeed, patients in poor clinical condition, with rapidly progressing disease and unresectable disease, were probably not offered this therapy. Thus, the patients who did receive the therapy probably had a better prognosis than the entire cohort of patients with recurrent osteosarcoma. However, the expected prognosis of our population remains poor because 38% had more than one previous relapse at the time of high dose TTP treatment, and about 50% of tumors were considered inaccessible for surgical resection. Nevertheless, 12 of 45 patients are alive without disease at 10 years, and 5-year OS is either equivalent or higher than in other published series. In the Cooperative Osteosarcoma Study Group (COSS) retrospective study reporting 576 cases of relapses treated with surgery and/or conventional chemotherapy, 5-year OS was 23% after the first relapse, and 5-year PFS was 13% after second relapse. Other published studies report equivalent survival rates, with reported median survival after relapse between 10 and 17 months [2–4, 36]. A recent report of the use of MTP with chemotherapy to treat metastatic recurrent osteosarcoma demonstrated a 4-year overall survival exceeding 30% in 165 patients with recurrent osteosarcoma, findings similar to ours [37]. Those patients underwent outpatient therapy and did not require intensive hospitalization or autologous stem cell support. Nevertheless, those authors did not clearly describe their inclusion criteria, and we cannot compare characteristics between their patients and ours. Moreover, in France, MTP is not currently approved by the health authorities and cannot be considered a therapeutic strategy available for relapsed osteosarcomas.

Finally, despite the fact that these patients previously underwent heavy chemotherapy, high dose thiotepa was well tolerated with no severe toxic event reported. This is an important point in our series.

## 5. Conclusion

In France, high dose thiotepa with PBSC rescue is proposed for the treatment of osteosarcoma relapses. In a population of patients with well-known poor prognosis, we observed a radiological response rate of 31% and 5-year OS of 31%. Our observation of relatively good radiological and histological response rates in this series and the relative safety of this strategy lead us to consider high dose thiotepa as an active drug in treatment of osteosarcoma. The French osteosarcoma group is engaged in a randomized study to compare the benefit of this treatment to conventional chemotherapy in relapsed osteosarcomas.

## Figures and Tables

**Table 1:** Patient and disease characteristics at initial diagnosis (*N* = 45).

	Number of patients	%
Patient characteristics		
Sex		
Male	29	64.4
Female	16	35.6
Characteristics of initial disease		
Localised disease	33	73.3
Lung metastasis	9	20.0
Bone metastasis	2	4.4
Lung + bone metastasis	1	2.2
Initial histological response		
Good response	17	37.8
Poor response	28	62.2
Initial treatment		
Surgery + chemotherapy	44	97.8
Surgery + chemotherapy + radiotherapy	1	2.2

**Table 2:** Patient characteristics at the time of relapse or progression treated with high dose thiotepa.

	Number of patients	%
Patient characteristics		
Age, years		
Median	15.9	
Range	4 to 31	
Disease status at study entry		
Refractory disease	2	4.4
First relapse	26	57.8
Subsequent relapse	17	37.8
Site of last relapse		
Lung only	23	51
Lung + another site	15	33
Bone only	1	2.2
Other	4	9.3
Relapse operability		
Operable	20	46.5
Inoperable	23	53.5
Missing value	2	
Radiological response		
Partial response	12	30.8
Stable disease	18	46.2
Progressive disease	9	23.1
Not evaluable	6	
Histological response after thiotepa		
Good response	9	29.0
Poor response	22	71.0
No surgery	14	

**Table 3:** Univariate analysis.

Factors	Number of patients	Number of deaths	5-year overall survival (%)	*P* value (Log-Rank test)
Initial histological response				
Good response	17	13	29	0.82
Poor response	28	20	17	
Radiological response				
Partial response	12	10	17	0.57
Stable disease or progressive disease	27	20	33	
Surgery after thiotepa (TTP)				
Yes	31	22	36	0.397
No	14	11	21	
Histological response after TTP				
Good response	9	2	56	0.17
Poor response	22	18	27	
Number of previous relapses				
First relapse	26	18	31	0.69
Subsequent relapse	17	13	29	
Delay between diagnosis and relapse before TTP				
≤2 years	19	16	16	0.01
>2 years	24	15	42	
